# Direct and indirect pro-inflammatory cytokine response resulting from TC-83 infection of glial cells

**DOI:** 10.1080/21505594.2018.1509668

**Published:** 2018-09-03

**Authors:** Forrest Keck, Stephanie Kortchak, Allison Bakovic, Brian Roberts, Nitin Agrawal, Aarthi Narayanan

**Affiliations:** a National Center for Biodefense and Infectious Diseases, George Mason University, Manassas, VA, USA; b Leidos Health Life Sciences, Frederick, MA, USA; c Krasnow Institute for Advanced Study, George Mason University, Fairfax, VA, USA

**Keywords:** Venezuelan equine encephalitis virus, microglia, astrocyte, pro-inflammatory cytokines, mitochondria

## Abstract

Venezuelan equine encephalitis virus (VEEV) is a neurotropic arbovirus that is highly infectious as an aerosol and can result in an encephalitic phenotype in infected individuals. VEEV infections are known to be associated with robust inflammation that eventually contributes to neurodegenerative phenotypes. In this study, we utilize the TC-83 strain of VEEV, which is known to induce the expression of IL-6, IL-8, and other pro-inflammatory cytokines. We had previously demonstrated that TC-83 infection resulted in changes in mitochondrial function, eventually resulting in mitophagy. In this manuscript, we provide data that links upstream mitochondrial dysfunction with downstream pro-inflammatory cytokine production in the context of microglia and astrocytoma cells. We also provide data on the role of bystander cells, which significantly contribute to the overall inflammatory load. Use of a mitochondrial-targeted antioxidant, mitoquinone mesylate, greatly reduced the inflammatory cytokine load and ameliorated bystander cell inflammatory responses more significantly than a broad-spectrum anti-inflammatory compound (BAY 11–7082). Our data suggest that the inflammatory mediators, especially IL-1β, may prime naïve cells to infection and lead to increased infection rates in microglial and astrocytoma cells. Cumulatively, our data suggest that the interplay between mitochondrial dysfunction and inflammatory events elicited in a neuronal microenvironment during a TC-83 infection may contribute to the spread of infection.

## Introduction

Venezuelan equine encephalitis virus (VEEV) is a neurotropic virus that causes periodic outbreaks of febrile and neurological disease in the Americas [1]. VEEV is an important human and equine pathogen that is transmitted by mosquitoes, with recent outbreaks involving thousands of human and equine cases spread over large geographical regions [1]. VEEV is a single-stranded, positive-sense RNA virus belonging to the genus *Alphavirus*, family *Togaviridae* [2,3]. Approximately 1% of all infections develop into severe encephalitic illness, which can result in coma and death. These deleterious outcomes are most closely associated with aerosol exposure, which allows the virus to quickly enter the brain via the olfactory nerve, where it can establish a robust infection [4–6]. Neuronal cells are highly permissive to VEEV infection, resulting in rapid viral dissemination, widespread neuroinflammation, and destruction of the blood brain barrier (BBB) [4]. Similar to other neurotropic viral infections, viremia in the brain is associated with long term neurological sequelae, including the potential for development of seizures [7–9]. This study utilizes the investigational TC-83 vaccine strain of VEEV, a live-attenuated virus that is known to induce a robust primary immune response and has been associated with severe side effects [10–12]. This underscores the need to understand the impact of inflammatory events elicited during infection, in order to design safe and effective intervention strategies.

The contribution of mitochondrial events to neuroinflammation has been extensively studied in the context of neurodegenerative diseases, and has recently been explored in the context of viral infections [13–23]. In these instances, alterations to mitochondrial homeostasis abrogate its function resulting in altered cellular redox status, accumulation of reactive oxygen species (ROS), dysregulated energy metabolism, mitophagy, increased neuroinflammation, collapsed mitochondrial networks, and axonal demyelination. We have previously reported that TC-83 can induce structural and functional changes to the mitochondria, which ultimately contributes to neuronal death [24].

It is well established that VEEV infection results in inflammation of the central nervous system. The attenuated TC-83 strain is known to induce pro-inflammatory cytokines such as interferon-γ (IFN-γ), interleukin-1β (IL-1β), IL-6, IL-8, IL-12, and tumor necrosis factor -α (TNF-α) [25–28]. These potent pro-inflammatory cytokines control several downstream targets which contribute to the inflammatory microenvironment. Several of these cytokines require glycogen synthase kinase-3β (GSK-3β) for production, a protein regulator that is indispensable for TC-83 replication. Therapeutics targeting GSK-3β offer protection against neurodegeneration in encephalitic VEEV infections and have also been shown to offer neuroprotection in Alzheimer’s disease [25]. This suggests that a pro-inflammatory environment may play a role in the establishment of infection in the brain, and emphasizes the importance of controlling neuroinflammation during viral infections.

Activation of the immune response by pathogen associated molecular patterns (PAMPs) is reliant on mitochondrial mechanisms for induction of pro-inflammatory cytokines [29–33]. These mechanisms are most prevalent in microglia, antigen-presenting neuroglia that scavenge the brain for insults and mediate several neuroinflammatory signaling events. Microglia-triggered inflammation has been documented to play significant roles in the progression of neurological disorders and viral infections [34–37].

In this study we utilize the TC-83 strain of VEEV to illustrate that microglia are susceptible to infection and that infection results in mitochondrial dysfunction in these cells. We determine that mitochondrial dysfunction contributes to the pro-inflammatory cytokines produced by direct infected and bystander activated microglia. Employing an antioxidant strategy effectively decreased these cytokine events, including IL-1β, which we implicate in increasing the infectivity of naïve bystander cells. The data that we present here reveals connections between upstream mitochondrial dysfunction, downstream pro-inflammatory cytokine production, and spread of viral infection in susceptible cells of neuronal origin in the context of TC-83 infection.

## Results

### Microglial cell lines are susceptible to VEEV infection

**Figure 1.**
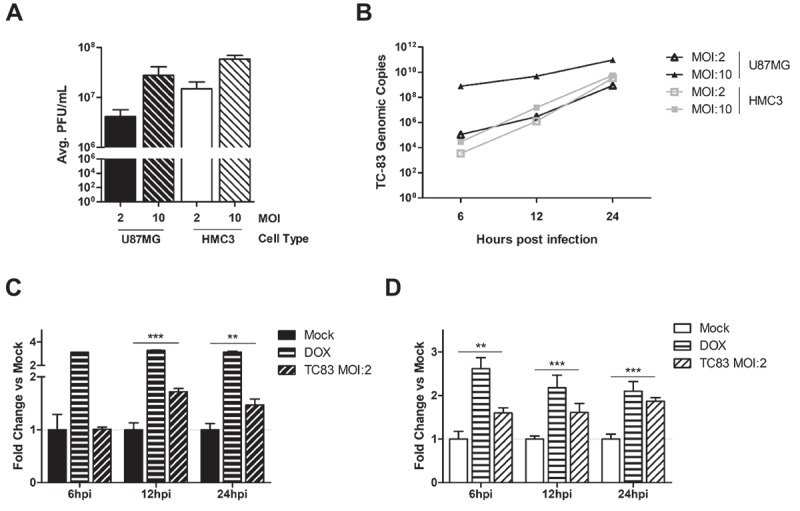
Glial cell lines sustain viral loads and induce caspase in a manner similar to the accepted astrocyte model of VEEV infection. U-87 MG astrocytes and HMC3 microglia were inoculated with TC-83 (MOI:2 or MOI:10). (a) Production of infectious virus at 24h was confirmed via plaque assay. (b) qRT-PCR confirmed that TC-83 can successfully replicate in both cell lines. Activation of caspase-3/-7 in (c) U-87 MG astrocytes and (d) HMC3 microglial cells infected with TC-83 (MOI:2). 100nM Doxorubicin (DOX) was used as a positive control for caspase-3/-7 induction. The quantitative data are depicted as the means of six biologically independent experiments ± SD. ** p < 0.01; *** p < 0.001.

Microglia, astrocytes, and neurons are integral components of the tissue microenvironment that is centrally involved in the development of VEEV-induced encephalitis. Our previously established U-87 MG astrocyte model was used as the standard for determining susceptibility of the HMC3 human-derived microglia to TC-83 infection. Quantification of infectious viral titers in these cell lines revealed that, while both cells experience a MOI-dependent increase in extracellular viral titers, U-87 MG cells produce slightly lower titers than HMC3 cells at 24 hours post infection (hpi) (Figure 1(a)). This difference is not the result of varying replication kinetics, as U-87 MG cells and HMC3 cells have equivalent production of TC-83 genomic copies at a MOI of 2 (Figure 1(b)).

Our previous study indicated that TC-83 induced mitochondrial dysfunction ultimately contributes to cell death via increased activation of the caspase response [24]. In astrocytes, the activation of caspase-3/7 is seen as early as 12 hours post infection (hpi) (Figure 1(c)). In microglia, cells were observed to experience increased caspase-3/7 activation as early as 6hpi (Figure 1(d)). These results indicate that TC-83 can infect microglial cells *in vitro* and produce cellular outcomes like those observed in astrocytoma cells.

### VEEV infection induces mitochondrial dysfunction in microglia

Studies investigating mitochondrial function in the context of viral infection have largely focused on production of ROS during viral insult. In addition to this accepted marker of mitochondrial stress, maintenance of the mitochondrial membrane potential (MMP) is also utilized as a marker of mitochondrial function [17,18,24,38]. We have shown that these two events correlate with increased mitochondrial fission and mitophagy, contributing to cell death in an infection dependent manner [24].

**Figure 2.**
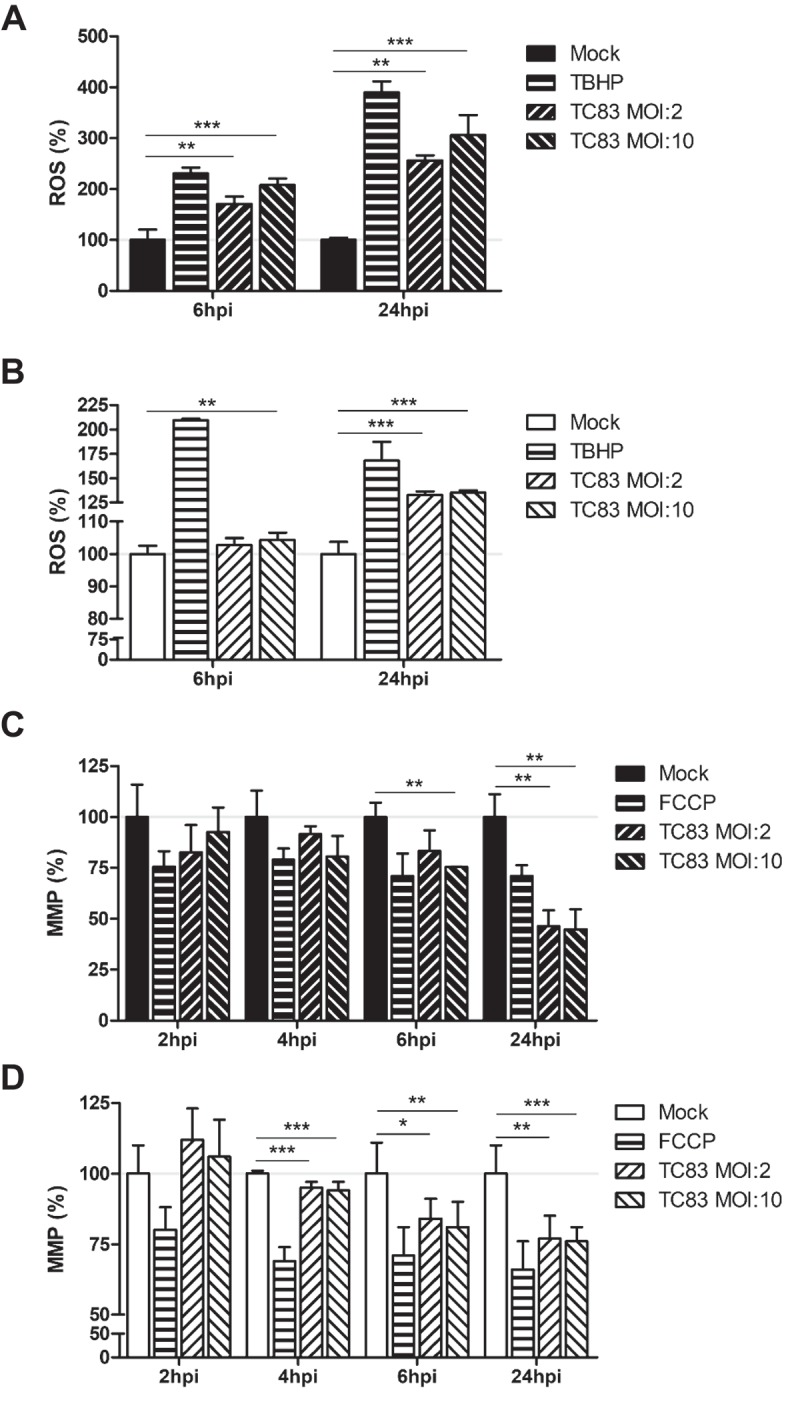
VEEV infection of microglial cells induces mitochondrial dysfunction. U-87 MG and HMC3 cells were inoculated with TC-83 (MOI:2 or MOI:10). Production of reactive oxygen species (ROS) was measured with 2ʹ,7ʹ-dichlorofluorescin (DCF) in infected (a) U-87 MG and (b) HMC3 cells. 100µM tert-butyl hydrogen peroxide (TBHP) was used as a positive control for ROS accumulation. Maintenance of the mitochondrial membrane potential (MMP) was measured with tetramethylrhodamine, ethyl ester (TMRE) in infected (c) U-87 MG and (d) HMC3 cells. 50µM carbonyl cyanide 4-(trifluoromethoxy) phenylhydrazone (FCCP) was a positive control for the disruption of MMP. The quantitative data are depicted as the means of six biologically independent experiments ± SD. * p < 0.05; ** p < 0.01; *** p < 0.001.

To assess whether similar mitochondrial dysfunction occurs in TC-83 infected astrocytes and microglia, we quantified the production of ROS and maintenance of the MMP as variables of infectious dose and time of infection. Six hours following infection (MOI:2), U-87 MG astrocytes experience a 171% increase in ROS, which increases to 256% by 24hpi (Figure 2(a)). Conversely, HMC3 microglia do not accumulate ROS until 24hpi, and to a lesser extent (133%) than observed in the astrocyte model (Figure 2(b)). The increased ROS in U-87 MG cells corresponds with a decrease in MMP, a 17% reduction at 6hpi and a 54% reduction at 24hpi (Figure 2(c)). The lessened accumulation of ROS in HMC3 cells correlated with a stronger maintenance of MMP, with a 16% reduction at 6hpi and a 23% reduction at 24hpi (Figure 2(d)). These time-dependent alterations to mitochondrial function indicate that microglia experience a loss of mitochondrial function following TC-83 infection.

### Infected microglia produce altered inflammatory cytokine profiles

**Figure 3.**
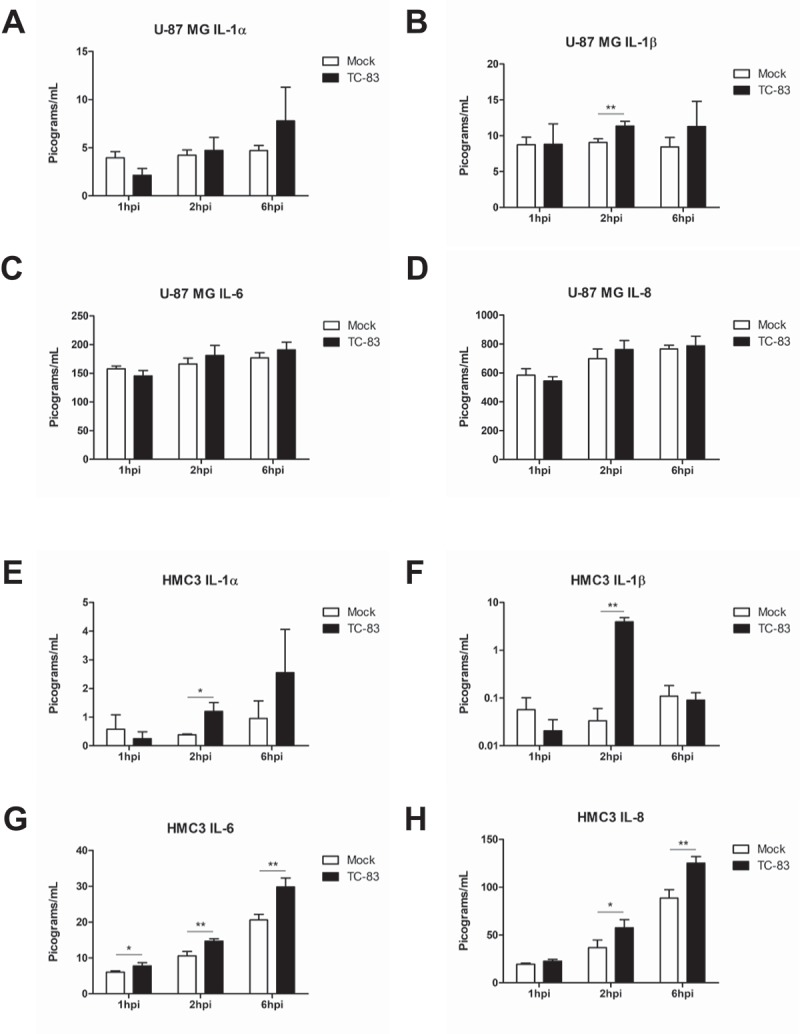
TC-83 induced inflammation in human astrocyte and microglial cells. (a-d) U-87 MG and (e-h) HMC3 cells were infected with TC-83 (MOI:2) and supernatants collected from these cells at 1, 2, and 6 hpi. Cytokine levels were determined for (a, e) IL-1α, (b, f) IL-1β, (c, g) IL-6, and (d, h) IL-8 in the context of direct TC-83 infection. The quantitative data are depicted as the means of three biologically independent experiments ± SD. * p < 0.05; ** p < 0.01.

It is widely accepted that microglia become activated following viral infection [39–43]. We observed that infected microglial cells follow the established activation profile after TC-83 infection (Supp. Figure 1). We hypothesized that upon activation, microglia would produce cytokines to induce a pro-inflammatory state. Using a 10-plex cytokine array, we analyzed U-87 MG astrocytes (Figure 3, top panels) and HMC3 microglia (Figure 3, bottom panels) infected with TC-83 for the production of inflammatory cytokines. Of the 10 cytokines queried (Supp. Figure 2), we focused our analysis on the pro-inflammatory cytokines IL-1α, IL-1β, IL-6, and IL-8, as these cytokines are critical to microglial immune function in the brain. In infected U-87 MG astrocytes, only the 2.31 pg/mL increase in IL-1β at 2hpi was significant (Figure 3(b)). Conversely, TC-83 infected HMC3 microglia display significantly increased levels of IL-1α and IL-1β at 2hpi (Figure 3(e,f)), increased IL-6 as early as 1hpi (Figure 3(g)), and increased IL-8 starting at 2hpi (Figure 3(h)). The increase in IL-1β from both cell types reveals a common pro-inflammatory mediator that could play a larger role in perpetuating neuroinflammation. Overall, this cell-type difference emphasizes the role that microglia play in modulating the pro-inflammatory cytokine response in the brain microenvironment.

### Bystander cells elicit a strong indirect pro-inflammatory cytokine response in the context of TC-83 exposure

**Figure 4.**
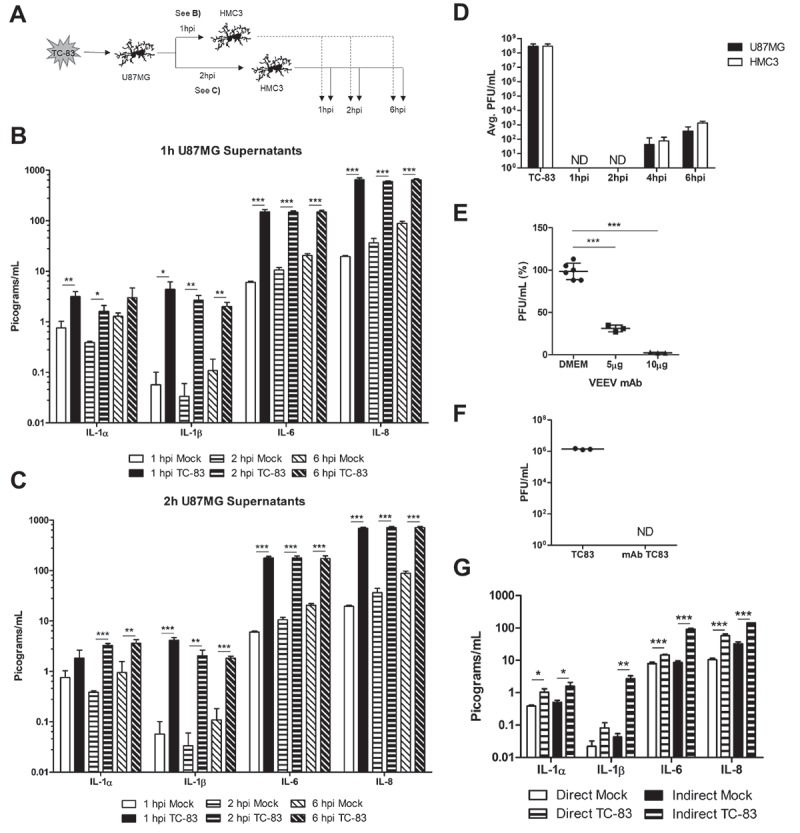
Cytokines produced by TC-83 infected astrocytes induce massive microglia-mediated pro-inflammatory storm. (a) Experimental workflow indicating U-87 MG cells were infected with TC-83 (MOI:2) for 1h, with supernatants removed at 1hpi and 2hpi. These supernatants were overlaid on naïve HMC3 microglia and inflammatory cytokines measured at 1, 2, and 6h post overlay. Cytokine analysis reveals induction patterns of IL-1α, IL-1β, IL-6, and IL-8 in (b) 1h U-87 MG supernatants, and (c) 2h U-87 MG supernatants. (d) Plaque assay indicates the time frame in which U-87 MG and HMC3 cells release infectious viral particles. (e) A monoclonal antibody was queried for its ability to neutralize VEEV input virus (MOI:2) for 30 minutes. Virus titers post-incubation were determined via plaque assay. (f) U-87 MG cells were infected with TC-83 for 1h, followed by the addition of media containing 10µg neutralizing antibody. Supernatants were removed at 6h post overlay and analyzed via plaque assay for infectious viral titers. (g) Cytokine analysis of (direct) TC-83 infected and (indirect) microglia receiving 1hpi U-87 MG supernatants in the context of 10µg neutralizing antibody. All cytokines were measured at 2hpi. (d, f) ND indicates virus was not detectable at these time points. The quantitative data are depicted as the means of three biologically independent experiments ± SD. * p < 0.05; ** p < 0.01; *** p < 0.001.

Our efforts are focused on understanding the cumulative impact of pro-inflammatory cytokines elicited by cells in the brain microenvironment. We hypothesized that pro-inflammatory cytokines produced as the result of bystander microglial cells interacting with infected astrocytes would play a critical role in contributing to the cytokine burden. As indicated by the experimental schematic (Figure 4(a)), supernatants were removed from U-87 MG cells that were infected with TC-83 and overlaid onto uninfected HMC3 cells. Using the cytokine array, we observed that the HMC3 cells overlaid with TC-83 infected U-87 MG supernatants from 1 h post infection (Figure. 4(b)) and 2(h) post infection (Figure 4(c)) displayed significantly increased levels of IL-1α, IL-1β, IL-6, and IL-8 when compared with the uninfected, time-matched control. IL-1α was most significantly upregulated in the 2h supernatants at 2h post overlay, with a 2.9 pg/mL increase over the mock. IL-1β, IL-6, and IL-8 were all most significantly upregulated in the 1h supernatants at 1h post overlay, with 4.3 pg/mL, 144.7 pg/mL, and 630.2 pg/mL increases over the 2h mock control.

To verify that the supernatants at those time points did not contain infectious virus, which could influence the production of pro-inflammatory cytokines, we performed plaque analysis on the supernatants from TC-83 infected cells at 1, 2, 4, and 6 hpi (Figure 4(d)). There was no detectable virus present in either U-87 MG astrocytes or HMC3 microglia at the 1h and 2h post infection time points. To further ensure that our noted cytokine profiles are not the result of virus present in the supernatants, we employed the use of a neutralizing monoclonal antibody specific to the envelope 2 glycoprotein of VEEV. At 10 µg, this antibody neutralized 97% of the input virus used for TC-83 infection (MOI:2) (Figure 4(e)). Using this antibody concentration, we repeated the overlay experiment from Figure 4(a), but instead added media containing neutralizing antibody following the 1h TC-83 infection. We then repeated our analysis of extracellular viral titers to confirm that no infectious virus was present in the supernatants (Figure 4(f)), and that the presence of antibody did not alter our expected cytokine profiles (Figure 4(g)). There was no significant difference in the direct or indirect cytokine levels reported with or without the monoclonal antibody control (Compare Figure 4(g) to Figure 3(e-h) & Figure 4(c)). These results indicate that microglia cells can be activated by cytokines produced from VEEV infected astrocytes in a manner that will contribute to the pro-inflammatory cytokine load.

### Primary astrocytes and microglia elicit similar direct and indirect pro-inflammatory cytokine responses

**Figure 5.**
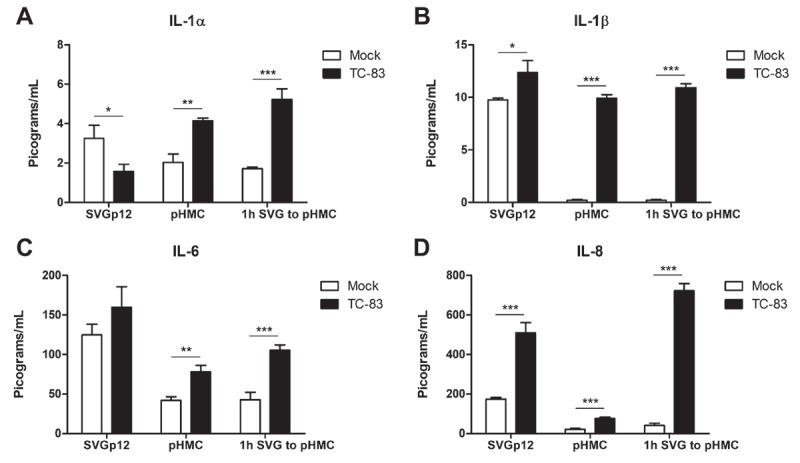
Pro-inflammatory response in direct infected and bystander primary cells. Primary human astrocytes (SVGp12) and primary human microglia (pHMC) were directly infected with TC-83 (MOI:2) and inflammatory cytokines analyzed at 2hpi. Separately, SVGp12 astrocytes were infected with TC-83 and supernatants transferred to naïve pHMC at 1hpi, with cytokine induction measured at 2h post overlay. Levels of the pro-inflammatory cytokines (a) IL-1α, (b) IL-1β, (c) IL-6, and (d) IL-8 are depicted as the means of six biologically independent experiments ± SD. * p < 0.05; ** p < 0.01; *** p < 0.001.

Our current efforts are focused on utilizing immortalized U-87 MG and HMC3 cell lines to characterize pro-inflammatory cytokine production in the context of TC-83 infection. To increase confidence in our model, we wanted to characterize the pro-inflammatory cytokine response elicited by primary human astrocytes (SVGp12) and primary human microglia cells (pHMC). We focused characterizing cytokine levels at 2hpi (TC-83 MOI:2), where we had the highest pro-inflammatory response in our immortalized cell line model. In SVG astrocytes, we observed a 1.7 pg/mL reduction in IL-1α (Figure 5(a)), a 2.6 pg/mL increase in IL-1β (Figure 5(b)), a 34.8 pg/mL increase in IL-6 (Figure 5(c)), and a 334.7 pg/mL increase in IL-8 signaling (Figure 5(d)). The primary astrocyte cell line had similar basal levels of IL-1α and IL-1β, but reduced quantities of IL-6 and IL-8.

In contrast to the U-87 MG model (Figure 3), the primary astrocytes experienced a significant drop in IL-1α at 2hpi, and a significant increase in IL-8. The primary microglia cells experienced a 2.1 pg/mL increase in IL-1α (Figure 5(a)), a 9.72 pg/mL increase in IL-1β (Figure 5(b)), a 35.93 pg/mL increase in IL-6 (Figure 5(c)), and a 55.09 pg/mL increase in IL-8 (Figure 5(d)) signaling at 2hpi during TC-83 infection. The pHMC cell line had similar basal levels of all cytokines to the immortalized HMC3 cells, but experienced two-fold more IL-1α, and five-fold more IL-6 cytokine induction at 2hpi.

In the context of the indirect activation of bystander pHMC cells by supernatants from TC-83 infected SVG astrocytes at 1hpi, we note significant increases in IL-1β, IL-6, and IL-8 that were statistically similar to the immortalized cell line data (Figure 4). Only IL-1α was differentially increased in the activated bystander pHMC cells, with 3-fold greater induction than the immortalized HMC3 bystander cells (Compare Figure 5(a) to Figure 4(b)). This data indicates that the immortalized cell line data accurately depicts the pro-inflammatory cytokine production induced in TC-83 infected primary astrocytes and microglia.

### Anti-inflammatory strategies rescue mitochondrial function in VEEV infected cells

**Figure 6.**
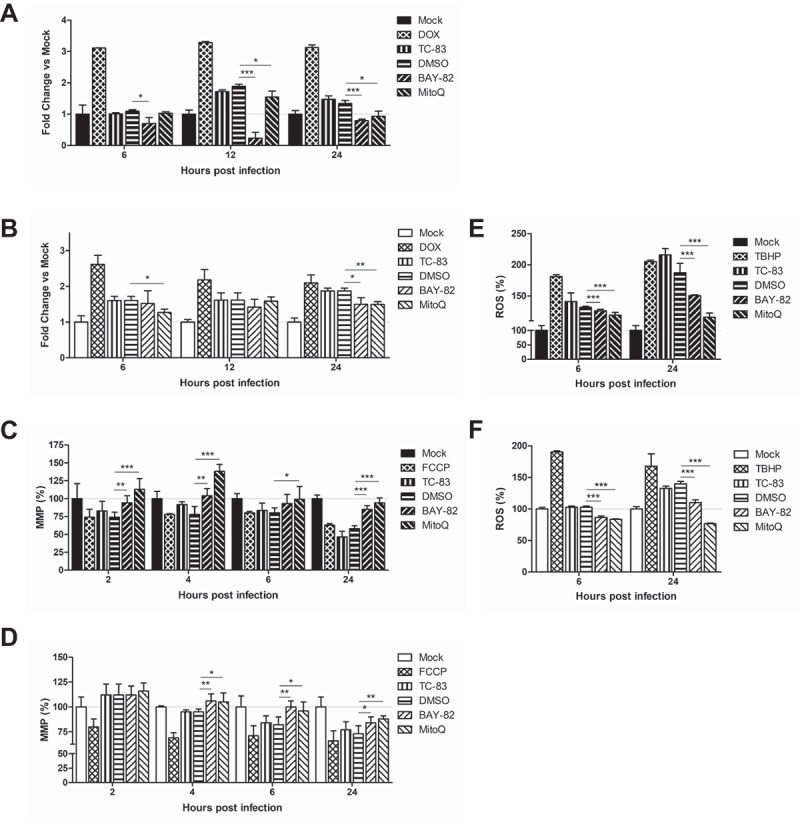
Anti-inflammatory intervention strategies are effective at mitigating mitochondrial damage during TC-83 infection. BAY 11–7082 (BAY-82) and mitoquinone mesylate (MitoQ) were analyzed for their ability to (a-b) decrease caspase-3/-7 induction, (c-d) rescue mitochondrial membrane potential, and (e-f) reduce production of reactive oxygen species in TC-83 inoculated (a, c, e) U-87 MG and (b, d, f) HMC3 cells. Controls: (a-b) Doxorubicin (DOX) was used as a positive inducer of the caspase-3/7 response. (c-d) Carbonyl cyanide 4-(trifluoromethoxy) phenylhydrazone (FCCP) is an ionophore uncoupler that induces MMP. (E-F) Tert-butyl hydrogen peroxide (TBHP) is a positive control for ROS accumulation. The quantitative data are depicted as the means of six biologically independent experiments ± SD. * p < 0.05; ** p < 0.01; *** p < 0.001.

We hypothesized that the induction of pro-inflammatory cytokines will be related to the mitochondrial dysfunction phenotype described in Figure 2. To this end, we utilized mitoquinone mesylate (MitoQ), a mitochondrially targeted antioxidant, to evaluate its impact on caspase-3/-7, MMP, and ROS in the context of TC-83 infection. We employed the NF-κB activation inhibitor BAY 11–7082 (BAY-82) as a control anti-inflammatory therapeutic strategy, and 0.1% dimethyl sulfoxide (DMSO) as our solvent control. We determined that the non-toxic dose was 1 µM for BAY-82 and 100pM for MitoQ, so all subsequent experiments were conducted using these concentrations. The antioxidant strategy was effective at reducing caspase-3/-7 induction in U-87 MG cells after 12hpi, while the anti-inflammatory reduced caspase-3/-7 at all time points (Figure 6(a)). In HMC3 cells, neither the anti-inflammatory BAY-82 nor the antioxidant MitoQ were effective at reducing the caspase activation at 12hpi, but each had a significant decrease in caspase activation at 24hpi (Figure 6(b)).

The antioxidant strategy was significantly more effective at restoring mitochondrial function, as MitoQ treated U-87 MG cells displayed up to 138% greater MMP than the uninfected control (Figure 6(c)). Cells receiving BAY-82 treatment were, on average, 20% less effective at recovering MMP than the antioxidant intervention. In HMC3 microglia, there was no difference between cells receiving MitoQ or BAY-82 treatment, as each therapeutic intervention was equally effective at increasing MMP after 4hpi (Figure 6(d)). The recovery of the MMP paralleled a decrease in ROS accumulation in the treated cells. BAY-82 and MitoQ were equally effective at reducing ROS burden at 6hpi in U-87 MG astrocytes, while MitoQ was most effective at eliminating ROS accumulation at 24hpi (Figure 6(e)). This trend recapitulated in HMC3 microglia, with both therapeutics equally capable of reducing ROS burden at 6hpi, but the antioxidant MitoQ more effective at 24hpi (Figure 6(f)). These results indicate that the mitochondrial accumulation of ROS during TC-83 infection contributes more significantly to the dysfunctional mitochondrial phenotype than NF-κB pro-inflammatory signaling.

### Mitochondrial dysfunction contributes to production of pro-inflammatory cytokines

**Figure 7.**
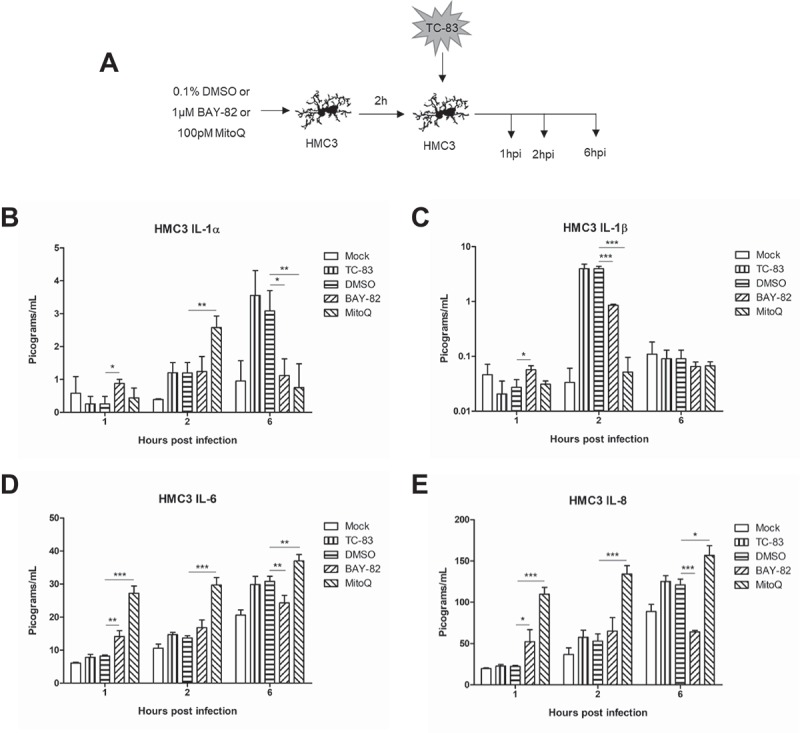
Controlling TC-83 induced ROS response in microglial cells decreases inflammatory burden. (a) Experimental workflow indicating HMC3 cells were pre-treated for 2h with 0.1% DMSO, 1μM BAY 11–7082 (BAY-82), or 100pM mitoquinone mesylate (MitoQ) prior to inoculation with TC-83 (MOI:2). Levels of the pro-inflammatory cytokines (b) IL-1α, (c) IL-1β, (d), IL-6 and (e) IL-8 produced by HMC3 cells as the direct result of TC-83 infection were measured at 1, 2, and 6hpi. The quantitative data are depicted as the means of three biologically independent experiments ± SD. * p < 0.05; ** p < 0.01; *** p < 0.001.

After observing the ability of MitoQ to restore mitochondrial function during TC-83 infection, we wanted to investigate how an antioxidant strategy would alter the direct pro-inflammatory cytokine production shown in Figure 3. U-87 MG astrocytes and HMC3 microglia were pre-treated with either BAY-82, MitoQ, or the solvent control prior to infection with TC-83 and subsequently analyzed for release of pro-inflammatory cytokines at 1, 2, and 6hpi (Figure 7(a)). We observed that MitoQ treatment leads to an initial increase in IL-1α at 2hpi, followed by a significant decrease at 6hpi (Figure 7(b)). MitoQ treatment also led to a significant decrease in IL-1β levels at 2hpi (Figure 7(c)). Interestingly, the antioxidant led to significantly more IL-6 and IL-8 at 1, 2, and 6hpi (Figure 7(d,e)). This result is directly correlated with the antioxidant, as BAY-82 treatment led to significantly decreased levels of IL-6 and IL-8 at 6hpi. These results support the possibility that mitochondrial dysfunction contributes to the microglial-mediated IL-1α/β pro-inflammatory cytokine production elicited in the context of TC-83 infection.

### The pro-inflammatory cytokine response elicited by bystander cells may be linked to mitochondrial dysfunction

**Figure 8.**
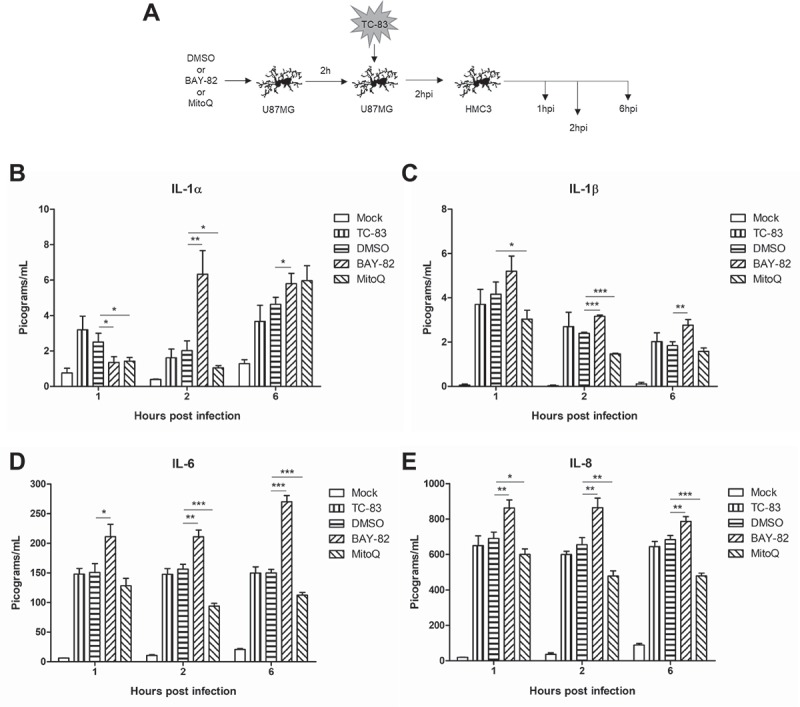
Indirect inflammatory response dependent on mitochondrially derived ROS. (a) Experimental workflow indicating that U-87 MG cells were pre-treated for 2h with 0.1% DMSO, 1µM BAY 11–7082 (BAY-82), or 100pM mitoquinone mesylate (MitoQ) prior to inoculation with TC-83 (MOI:2). Supernatants were removed from infected U-87 MG cells at 2hpi and overlaid onto naïve HMC3 microglia, with (b) IL-1α, (c) IL-1β, (d) IL-6, and (e) IL-8 cytokine levels measured at 1, 2, and 6 hours post overlay. The quantitative data are depicted as the means of three biologically independent experiments ± SD. * p < 0.05; ** p < 0.01; *** p < 0.001.

We hypothesized that a strategy that will control ROS accumulation and restore MMP will also decrease the pro-inflammatory cytokine burden elicited by the bystander cells, as characterized in Figure 4. To investigate this phenomenon, we treated U-87 MG astrocytes with DMSO, BAY-82. or MitoQ, infected those cells with TC-83. Supernatants were then removed from infected cells at 2hpi and overlaid onto uninfected HMC3 cells (Figure 8(a)). The uninfected cells were queried for cytokine production at 1, 2, and 6 hours post inoculation. In the context of BAY-82 pre-treated U-87 MG overlays, there was a marked increase in IL-1α and IL-1β at 2 and 6hpi, and in IL-6 and IL-8 at all time points (Figure 8(b-e)). Contrastingly, the antioxidant therapy MitoQ reduced levels of IL-1α at 1 and 2hpi (Figure 8(b)), which corresponded with a decrease in IL-1β at the same time points (Figure 8(c)). The marked increase in IL-6 and IL-8 noted in directly infect HMC3 microglia was not seen in naïve microglia receiving U-87 MG supernatants, with significant decreases in IL-6 (Figure 8(d)) and IL-8 (Figure 8(e)) observed at all time points. This suggests that reducing oxidative stress in the astrocytes will more greatly contribute to reducing the pro-inflammatory cytokine burden than targeting infected microglia directly. Overall, these results support the possibility that mitochondrial dysfunction could play a role in the inflammatory cytokine responses elicited by bystander cells during TC-83 infection.

### Pro-inflammatory cytokines increase spread of TC-83 infection

**Figure 9.**
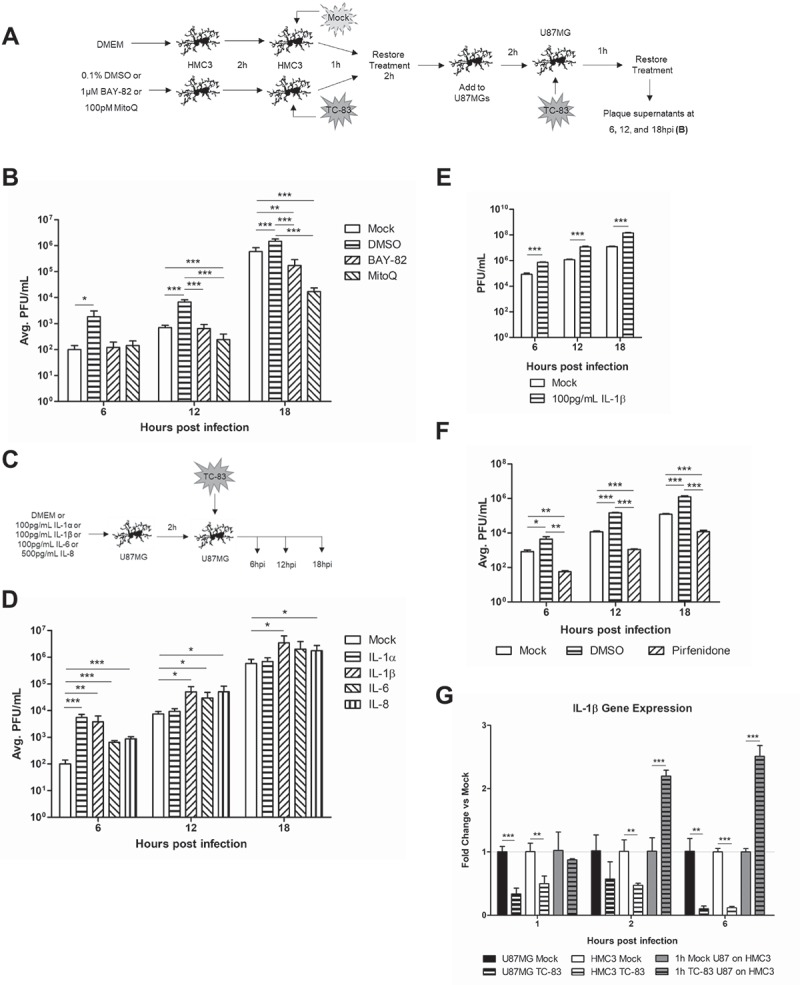
IL-1β released by infected microglial cells prime astrocytes for increased TC-83 uptake. (a) Experimental workflow indicating the pre-treatment and infection schematic for HMC3 cells. Supernatants were removed from TC-83 inoculated HMC3 cells and overlaid onto naïve U-87 MG cells prior to inoculation of the astrocytes with TC-83 (MOI:2). (b) Production of infectious virus in these treatment conditions was measured at 6, 12, and 18hpi by plaque assay. (c-d) To determine which pro-inflammatory cytokines are contributing to the increase in TC-83 uptake, astrocytes were pre-treated with biologically relevant doses of IL-1α, IL-1β, IL-6, or IL-8 and production of infectious virus measured at 6, 12, and 18hpi by plaque assay. (e) Supernatants from HMC3 cells pre-treated with 100pg/mL IL-1β were overlaid on naïve U-87 MG astrocytes prior to TC-83 infection (MOI:2). Plaque assay measured production of infectious virus at 6, 12, and 18hpi. (f) The infection scheme from (a) was repeated in the context of 100µM Pirfenidone, a potent IL-1β inhibitor. (g) IL-1β gene expression was measured in the context of direct infected U-87 MG and HMC3 cells, and 1h supernatants from infected U-87 MG cells overlaid on naïve HMC3 microglia. Gene induction was determined via the ΔΔCτ method with 18S as the endogenous control. The quantitative data are depicted as the means of three independent experiments ± SD. For all images, representative data were selectively obtained from three individual experiments. * p < 0.05; ** p < 0.01; *** p < 0.001.

We have demonstrated that mitochondrial events may exacerbate the pro-inflammatory cytokine response elicited by bystander cells, thus increasing the overall inflammatory cytokine load. We suggest that the cytokines being released by infected HMC3 microglia may prime surrounding cells to become more susceptible to infection and sustain a higher viral load than a cell that was not primed by such an inflamed environment. To this effect, we pre-treated and infected HMC3 microglia with TC-83 and then added the supernatants obtained at 2h post infection to naïve U-87 MG cells (Figure 9(a)). These U-87 MG cells were then infected with TC-83 and extracellular viral titers were determined via plaque assay at 6, 12, and 18hpi (Figure 9(b)). We observed that U-87 MG cells that were pre-treated with supernatants from infected HMC3 microglia produced ten times more virus than astrocytes overlaid with supernatants from uninfected HMC3 microglia. Astrocytes receiving supernatants from BAY-82 conditioned microglia did not display this increase in infectivity, with titers matching the mock control at 6hpi and 12hpi and producing significantly less virus at 18hpi. Astrocytes receiving supernatants from MitoQ conditioned microglia produced significantly less virus than mock-treated cells starting at 12hpi, leading to an overall 2 log reduction in viral titers at 18hpi. A possible explanation of this phenomenon is that the pro-inflammatory mediators in the supernatant mediate the increase in infectivity.

**Figure 10.**
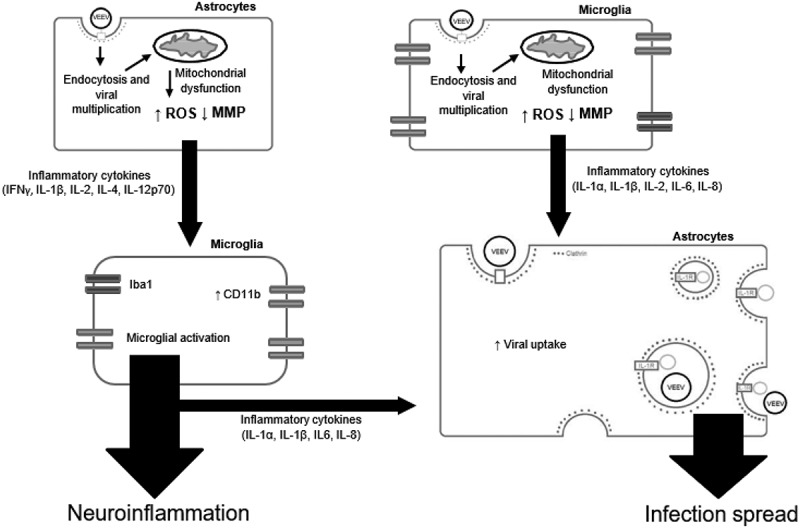
A hypothetical model of VEEV-induced neuroinflammatory environment. VEEV can directly infect astrocytes and microglia, leading to an accumulation of dysfunctional mitochondria. These mitochondrial events drive inflammatory cytokine signaling, which activate bystander microglia and further promote a pro-inflammatory environment. Continued induction of this inflammatory microenvironment leads to an increase in viral uptake, which may be dependent on IL-1β. Cumulatively, these events promote the spread of VEEV infection in the brain.

To further elucidate the impact the pro-inflammatory mediators had on the astrocyte’s susceptibility to infection, we added purified inflammatory cytokines to the supernatants of astrocytes prior to TC-83 infection (Figure 9(c)). The cytokine doses used in this experiment were determined from the inflammatory mediator increases noted in Figure 4 (100 pg/mL of either IL-1α, IL-1β, or IL-6, or 500 pg/mL of IL-8). U-87 MG cells pre-treated with IL-1α had the most significant increase in viral titers at 6hpi, while IL-6 and IL-8 pre-treatment increased viral titers most significantly at the later time points (Figure 9(d)). Cells treated with IL-1β produced the most virus at all of time points examined. This lead us to suggest that IL-1β might play the most significant role in priming naïve astrocytes for TC-83 infection and contribute to the spread of the infection in the neuronal microenvironment. To further explore this hypothesis, we repeated the treatment and infection schematic from Figure 9(a) using 100 pg/mL of IL-1β instead of a therapeutic. We observed that when pre-treated with IL-1β, microglia produce inflammatory mediators that significantly increase the viral uptake of bystander astrocytes, with a ten-fold increase in viral titers over the mock control (Figure 9(e)). Use of the IL-1β inhibitor Pirfenidone in the context of the infectivity experiment reveals significantly reduced viral titers at all time points queried, indicating that IL-1β pro-inflammatory signaling is significantly contributing to the priming of the surrounding microenvironment (Figure 9(f)). While we anticipate that caspase-1 mediated activation of pro-IL-1β will contribute to activated IL-1β levels in infection scenarios, we expect that a portion of the IL-1β produced will be the result of increased gene expression. We note that in direct infected U-87 MG and HMC3 cells, IL-1β gene expression is significantly inhibited (Figure 9(g)). This corresponds with viral capsid protein-mediated inhibition of host transcription previously reported in TC-83 infection [44]. Most importantly, HMC3 cells receiving overlays from infected U-87 MG astrocytes display a 2-fold induction of IL-1β gene expression as early as 2hpi. This further implicates IL-1β as a potential mediator of the increased infectivity phenotype.

## Discussion

Infection of neuronal tissue by neurotropic viruses is often associated with a significant level of inflammation, which has also be linked to BBB permeability and long-term neurological consequences. Thus, it is undeniable that collateral damage resulting from inflammatory events elicited in bystander cells plays an important role in neurodegenerative outcomes during a viral infection. It is also highly likely that the inflammatory dynamics and ensuing tissue damage will not be abrogated by antiviral strategies alone. Successful interventions will have to address inflammatory events elicited by infected and bystander cells in order to protect the host from long-term neurological damage.

Our data indicates that *in vitro*, human microglia (HMC3 cells) are infected by the TC-83 strain of VEEV in a manner that leads to caspase-3/-7 activation, mirroring the accepted U-87 MG astrocyte model. Upon infection, both the astrocytoma and microglial cell lines experience an accumulation of ROS in a manner that parallels decreased mitochondrial membrane potential. This mitochondrial dysfunction is more pronounced in the astrocytoma model, which we suggest is the result of cell-type specific tolerances to mitochondrial damage. Scavenging microglia could have a higher tolerance to ROS accumulation that allow it to sustain an equivalent viral load to astrocytes without experiencing similar mitochondrial consequences.

It is well accepted that microglia become activated during neurotropic viral infections, with activation leading to the expression of numerous downstream pro-inflammatory responses. In the context of TC-83 infection, we note that U-87 MG astrocytes produce increased levels of IL-1β, while HMC3 microglia release more IL-1α, IL-1β, IL-6, and IL-8. We observed similar alterations in TC-83 infected primary astrocytes and primary microglia, suggesting that our *in vitro* model can accurately depict inflammation induced in primary cell cultures. As astrocytoma cells experienced more pronounced mitochondrial dysfunction phenotypes, the relationship between mitochondrial dysfunction and inflammation in infected cells appears to be non-linear. This difference in pro-inflammatory cytokines is most likely the result of cell-specific functions, with the microglia’s role in leukocyte recruitment imparting a stronger inflammatory cytokine response to these cells than astrocytes.

Given the scavenging role of microglia, we acknowledge that our *in vitro* characterization of direct infected microglia may not accurately reflect a real-life infection scenario. *In vivo*, it is more likely that scavenging microglia encounter an infected astrocyte or neuron and become activated by signals released from these more permissive cell types. Activation of bystander microglia is strongly linked with neuroinflammation in a number of neurodegenerative diseases, and we hypothesized that it could significantly contribute to the pro-inflammatory cytokine load during VEEV infection [45,46]. Our investigation revealed that bystander microglia exposed to overlays from infected astrocytes produce significantly more IL-1α, IL-1β, IL-6, and IL-8 as compared to the directly infected cells. These pro-inflammatory cytokines are strongly linked with BBB damage, which indicates that BBB permeability may be the result of bystander cell inflammation rather than direct infected cells [47–49]. This cytokine induction recapitulates in our primary cell model and occurs as early as 1 hour post infection, which underscores the need for an effective therapeutic that can mitigate this massive pro-inflammatory cytokine response.

Since accumulation of ROS is known to contribute to inflammation, we anticipated that use of a mitochondria-directed antioxidant would decrease neuroinflammation in the context of VEEV infection. We found that astrocytes and microglia treated with the antioxidant MitoQ prior to TC-83 infection experienced decreased accumulation of ROS and increased MMP, corresponding with reduced caspase-3/-7 activation. The antioxidant was more effective at recovering cellular functions than the traditional anti-inflammatory (BAY-82), indicating that mitochondrial-derived ROS play a significant role in perpetuating negative cell outcomes during infection. We also reveal that the antioxidant therapy decreases levels of IL-1α and IL-1β produced by direct infected microglia, and decreases levels of IL-1α, IL-1β, IL-6 and IL-8 produced as the result of bystander activation. This suggests that mitochondrial dysfunction is a significant contributor to the net pro-inflammatory cytokine load elicited as the result of TC-83 infection. Interestingly, MitoQ treatment prior to direct TC-83 infection of microglia corresponded with increased IL-6 and IL-8, while treatment with BAY-82 in both direct and indirect infection scenarios led to increased levels of all cytokines queried. This data suggests that recovery of mitochondrial function be dependent on up-regulation of IL-6 and IL-8. This emphasizes the need to mitigate mitochondrial stress in order to decrease microglia-mediated neuroinflammation in the context of VEEV infection.

Given the profound number of intercellular interactions that exist in the brain microenvironment, we reasoned that cross-cellular impact of infected and bystander cells is likely to play a role in the spread of viral infection. Our current work indicates that the pro-inflammatory cytokine load, particularly IL-1β, is likely to push naïve cells to a more susceptible phenotype, with bystander cells sustaining higher viral load than naïve cells, particularly during the early stages of infection. Given the observed decrease in IL-1β with MitoQ intervention, we suggest that accumulated ROS contributes to the induction of IL-1β in the context of bystander activation. Ongoing efforts in our laboratory are directed at further elucidating the role of IL-1β in the increased infectivity phenotype.

As summarized in Figure 10, our data suggest that viral-mediated mitochondrial damage is likely to be a critical component to the establishment of the neuroinflammatory microenvironment. This pro-inflammatory signaling is the result of both infected and bystander cells and cumulatively contributes to the spread of infection in the brain. Our data further implicate the mitochondria as a broadly applicable therapeutic target for treating neurotropic infections in a manner that serves the dual purpose of stemming pro-inflammatory responses and controlling the spread of the infection.

## Materials and methods

### Antibodies, inhibitors, and reagents

The reagents and antibodies (Abs) used were as follows: dimethyl sulfoxide (DMSO) (VWR 97,063–136); the mitochondrially targeted antioxidant mitoquinone mesylate (MedKoo Biosciences 317,102); the NF-κB inhibitor BAY 11–7082 (Selleckchem S2913); the IL-1β inhibitor Pirfenidone (SelleckChem S2907); IL-1α, IL-1β, IL-6, and IL-8 recombinant protein (Aushon 101-3FF-1-AB); mouse monoclonal Ab to TOMM20 (Abcam ab56783), Alexa Fluor 488-conjugated goat anti-mouse (Invitrogen A21202), Alexa Fluor 568-conjugated donkey anti-goat (Invitrogen A11057); Anti-Venezuelan equine encephalitis antibody clone 1A3B-7 (EMD Millipore MAB8755); and polyclonal goat serum to TC-83 capsid protein (BEI Resources NR-9403).

### Viruses and cell lines

The live-attenuated virus (TC-83) used in this study was obtained from BEI Resources. TC-83 was attenuated from the fully virulent Trinidad Donkey (TrD) strain in a manner previously described [2]. U-87 MG human astrocytoma cells (ATCC HTB-14), SVGp12 primary human astrocytes (ATCC CRL-8621), VERO African green monkey kidney cells (ATCC CCL-81), and HMC3 human microglial cells (ATCC CRL-3304) were obtained from the American Type Culture Collection. Primary CNS cortex human microglial cells (37,089–01) were obtained from Celprogen. U-87 MG and VERO cells were grown in Dulbecco’s Modified Eagle’s Medium (DMEM) with 4.5 g/L Glucose, 2mM L-glutamine (VWR Life Science VWRL0102-500), 10% heat-inactivated fetal bovine serum (FBS) (Gibco 10,437,028), 10 U/ml of penicillin and 10 μg/ml of streptomycin (Gibco 15–140-122). HMC3, SVGp12, and primary microglial cells were grown in Eagle’s Minimum Essential Medium (EMEM) (ATCC 30–2003) with 10% heat-inactivated FBS, 10 U/ml of penicillin and 10 μg/ml of streptomycin. All cells were grown in a humidified incubator set to 37.1°C with an atmosphere of 5% CO_2_ and 95% air.

### VEEV infection and plaque assay

Cells were seeded at a density of 10 [4] cells per 100 µl in 96-well plates overnight, followed by the addition of DMEM with TC-83 (MOI of 2). The cells were incubated for 1h at 37.1°C. Then, the cells were washed once with medium and incubated at 37.1°C with 5% CO_2_ for the required interval. The viral supernatants were checked using plaque assays. For the plaque assay, Vero cells were plated in 12-well plates at a density of 2.5 x 10 [5] cells/well and cultured in DMEM at 37.1°C, 5% CO_2_. After a 1h adsorption with a serially diluted virus solution, wells were covered with Eagle’s Minimum Essential Medium (Quality Biological 115–073-101) (without phenol red, supplemented with 10% FBS, non-essential amino acids, 1 mM sodium pyruvate, 2 mM L-glutamine, 20 U/ml of penicillin and 20 μg/ml of streptomycin) containing 0.6% agarose (Life Technologies). Two days post-infection, the cells were fixed with 10% formaldehyde for 1h, the medium was removed, and cells were stained with a crystal violet solution containing 1% crystal violet, and 20% ethanol.

### Cell viability

Cell viability was assessed using the CellTiter Glo assay (Promega G7570), according to the manufacturer’s instructions. Viable cells produced ATP, which provided the energy to convert the inactive luciferin to oxyluciferin, which was then detected using a microplate reader. Luminescence was read using a Beckman Coulter DTX 880 multimode plate reader, with an integration time of 0.5s per well.

### Quantitative real-time polymerase chain reaction

*Viral RNA*. Cells were lysed, and viral RNA extracted using the QIAamp Viral RNA kit (Qiagen 52,906) as per the manufacturer’s instructions. Viral RNA was quantitated using qRT-PCR with primers and probe targeting the capsid sequence (nucleotides 7931–8005) of VEEV TC-83 [25]. qRT-PCR pre-cycling conditions were in accordance with manufacturer’s instructions for the Verso 1-step RT-qPCR kit (ThermoFisher AB4101C). 40 amplification cycles were performed, with an annealing temperature of 61°C. The primer pairs were originally described by Julander: forward primer (TCTGACAAGACGTTCCCAATCA) and reverse primer (GAATAACTTCCCTCCGACCACA). The Julander described 5ʹ 6-FAM/3ʹ TAMRA probe was adapted to a 5ʹ 6-FAM/ZEN/3ʹ IBFQ probe with the following sequence to improve sensitivity (5ʹ6-FAM/TGTTGGAAG/ZEN/GGAAGATAAACGGCTACGC/3ʹIABkFQ). Absolute quantification was calculated based on the threshold cycle (Ct) relative to the standard curve. *IL-1β*. Cells were lysed using 300 µL TRIzol reagent, and total RNA extracted using the Zymo Direct-zol MagBead kit (Zymo Research R2104) as per the manufacturer’s instructions. The total RNA was then converted to cDNA using a high-capacity cDNA reverse transcription kit (Thermo Fisher 4,368,814). Expression of IL-1β was determined using the ΔΔCτ method with Taqman reagents for IL-1β (Thermo Fisher HS01555410_m1) with 18S as the endogenous control (Thermo Fisher Hs99999901_s1).

### Caspase-3/-7 detection assay

Caspase-3/-7 activity was quantitated using the Caspase-Glo 3/7 Assay (Promega G8090) according to the manufacturer’s instructions. The Beckman Coulter DTX 880 multimode plate reader was used to query the processed samples.

*Cellular reactive oxygen species detection*. This assay utilized the cell permeant, fluorogenic dye 2ʹ,7ʹ-dichlorofluorescin diacetate (DCFDA) to measure cellular production of reactive oxygen species (ROS). In the cell, DCFDA is deacetylated by cellular esterases to a non-fluorescent form, which is later oxidized by ROS into the highly fluorescent 2ʹ,7ʹ-dichlorofluorescin (DCF). The excitation/emission spectrum of DCF is 495/529nm, respectively, which was measured using at Ex/Em 485/535 on a Beckman Coulter DTX 880 multimode reader. This assay was obtained from Abcam (ab113851) and carried out according to the manufacturer’s instructions. As a positive control, cells were treated with 100 µM tert-butyl hydrogen peroxide (TBHP) to induce DCF fluorescence.

### Mitochondrial membrane potential assay

This assay quantified mitochondrial membrane potential (MMP) in live cells by microplate spectrophotometry. Using the cell permeant, positively-charged, red-orange dye tetramethylrhodamine, ethyl ester (TMRE), active mitochondria can be queried based on accumulation of TMRE in the negatively charged membrane. Once mitochondria became depolarized or inactivated, they can no longer sequester TMRE, which is measured as decreased fluorescence. As a positive control, cells were treated with 50 µM carbonyl cyanide 4-(trifluoromethoxy) phenylhydrazone (FCCP), which is an ionophore uncoupler of oxidative phosphorylation that eliminated MMP and TMRE staining. This assay was obtained from Abcam (ab113852) and carried out according to the manufacturer’s instructions. TMRE was measured on a Beckman Coulter DTX 880 at Ex/Em 549/575 nm.

### Antibody neutralization

U-87 MG astrocytes were infected with TC-83 (MOI:2) for 1 hour. Following infection, supernatants were removed, and cells were washed 2X with PBS. Media containing 10 µg of VEEV monoclonal antibody (EMD Millipore MAB8755) was then added to the astrocytes. 2 hours post overlay, supernatants were removed and subjected to either plaque assay analysis of infectious viral titers or analyzed via inflammatory cytokine array.

### Inflammatory cytokine array

To detect cytokines in cell culture supernatants, samples were processed using the human 10-plex Ciraplex array (Aushon 101-3FF-1-AB) according to the manufacturer’s instructions. The Aushon Cirascan ASP-2010 was used to image the immunoassay. Cirascan images were analyzed using the Cirasoft Analysis Software according to the 101-3FF-1-AB kit alignment. Analyte concentrations were converted to fold change over time matched controls using Prism 5 software.

### Statistical analysis

The software Prism 5 (Graph Pad) was used for all statistical analyses. Data are presented as mean ± SD. When appropriate, the number of replicates depicted by each graph is indicated in the figure legend. Results were analyzed by unpaired, two-tailed t-test. Differences in statistical significance are indicated with asterisks: * p < 0.05; ** p < 0.01; *** p < 0.001.

## Abbreviations

BAY-82: anti-inflammatory molecule BAY-11–7082
BBB: blood brain barrier
DCFDA: 2ʹ,7ʹ-dichlorofluorescin diacetate
FCCP: carbonyl cyanide 4-(trifluoromethoxy) phenylhydrazone
GSK-3β: glycogen synthase kinase-3β
IFN: interferon
IL: interleukin
JEV: Japanese encephalitis virus
MitoQ: mitoquinone mesylate
MMP: mitochondrial membrane potential
RNA: ribonucleic acid
ROS: reactive oxygen species
RT-PCR: real time polymerase chain reaction
TBHP: tert-butyl hydrogen peroxide
TC-83: attenuated strain of Venezuelan equine encephalitis virus
TMRE: tetramethylrhodamine, ethyl ester
TNFα: tumor necrosis factor α
VEEV: Venezuelan equine encephalitis virus
