# Supplementary material for: Direct and indirect pro-inflammatory cytokine response resulting from TC-83 infection of glial cells
Source: Virulence. 2018 Sep 3;9(1):1403–21. doi: 10.1080/21505594.2018.1509668 (PMC6141141; doi:10.1080/21505594.2018.1509668)
Supplement: Supplemental Material [file kvir-09-01-1509668-s001.pdf]

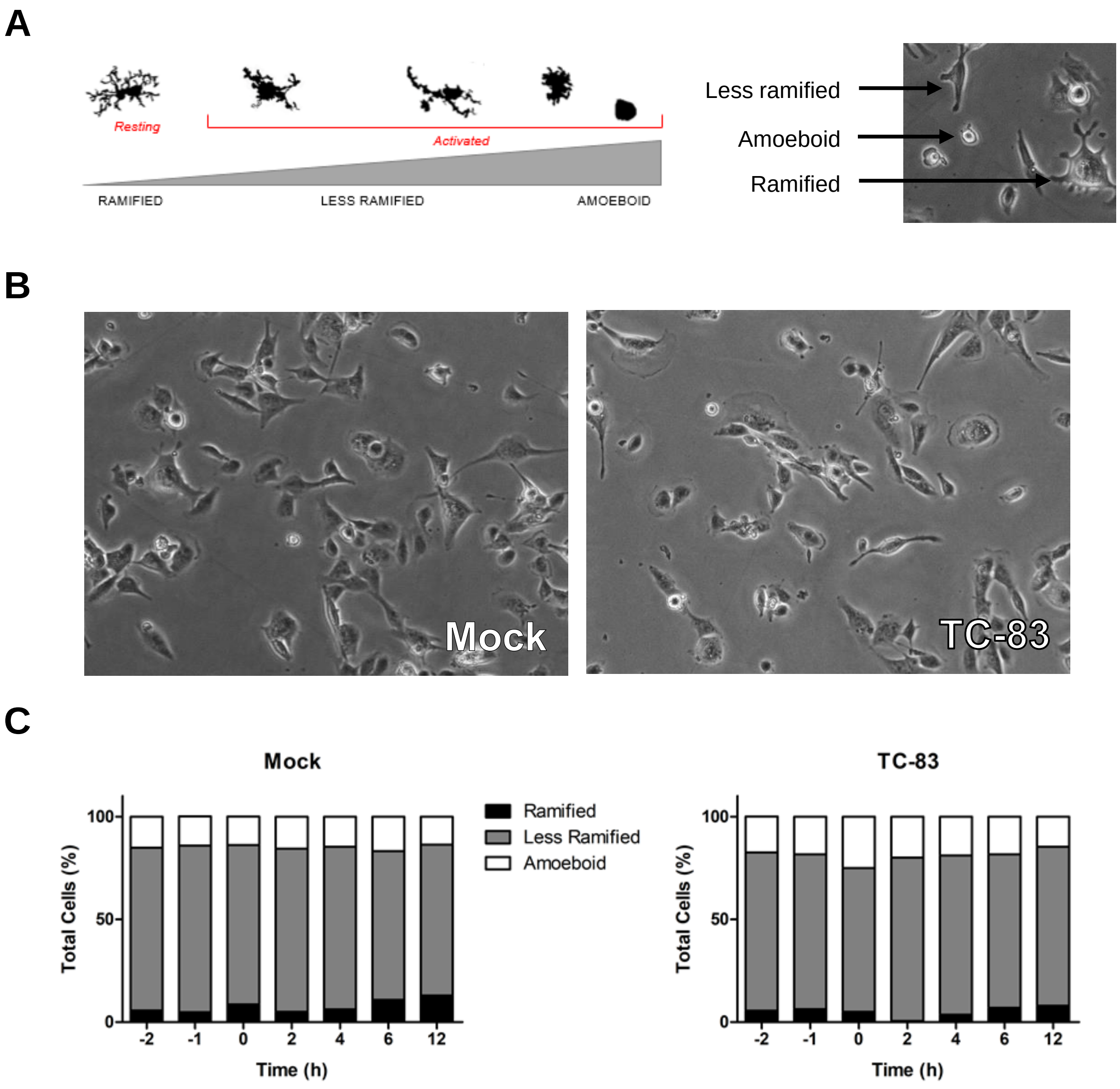


***Supplemental Figure 1.*** HMC3 microglia cells become activated as the result of VEEV infection. (A) Representative diagram of microglial morphology corresponding to activation. Examples of these morphologies are indicated in the context of our time lapse microscopy. (B) Time lapse microscopy shows changes in HMC3 cell morphology during infection with TC-83 (MOI:2) characterized as ramified, less ramified, and amoeboid. (C) The quantitative data for each experimental condition are shown as percentage of total cell count from three biologically independent experiments.

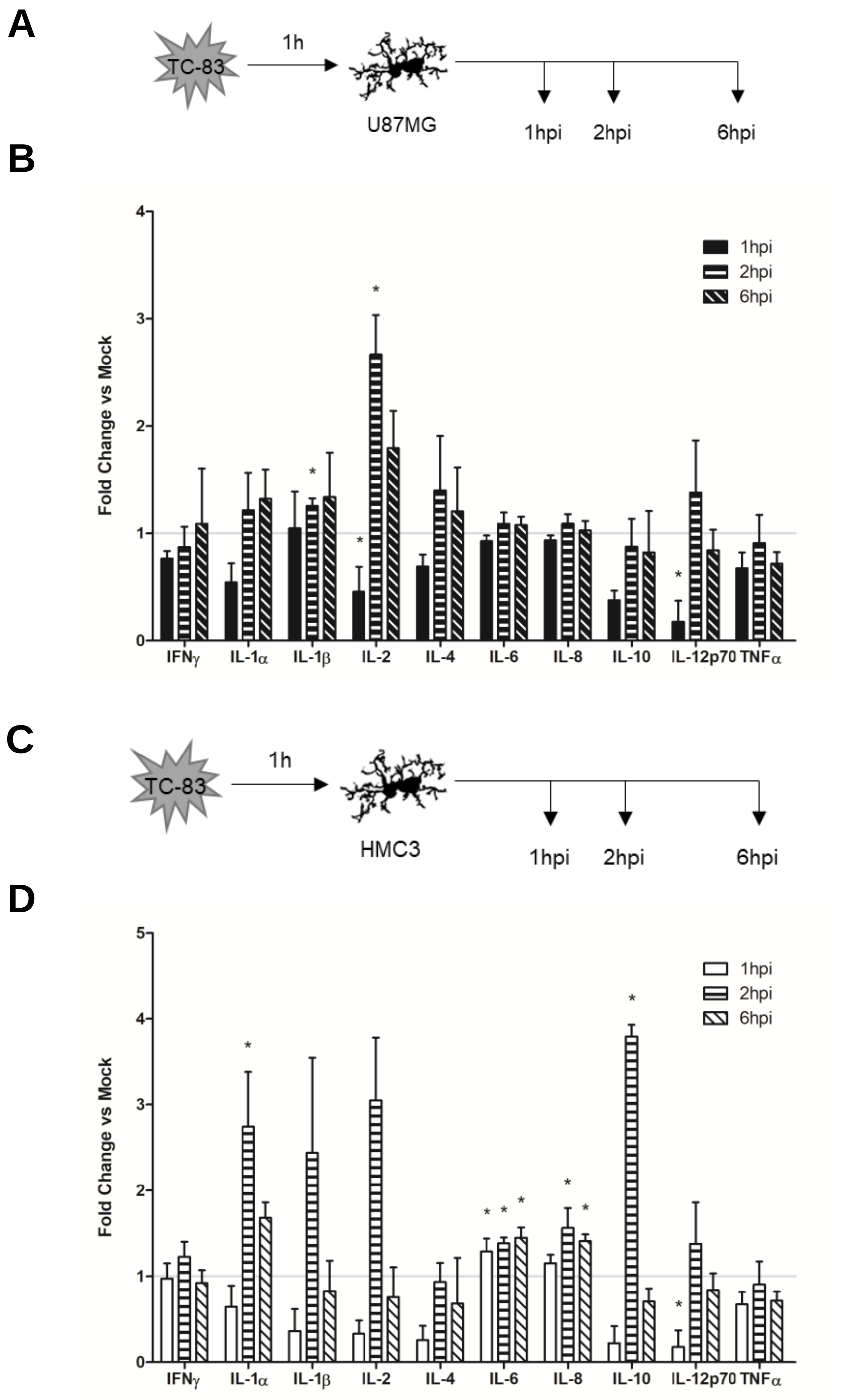


***Supplemental Figure 2*.** TC-83 induced inflammation in human astrocyte and microglial cells. Experimental workflow for the infection and collection of supernatants from (A) U-87 MG and (C) HMC3 cells. Inflammatory cytokine profiles are displayed for TC-83 (MOI:2) infected (B) U-87 MG and (D) HMC3 cells. Cytokines are depicted as fold induction as compared to cell-type matched, time-dependent controls. The quantitative data are depicted as the means of three biologically independent experiments ± SD. *$p < 0.05$.

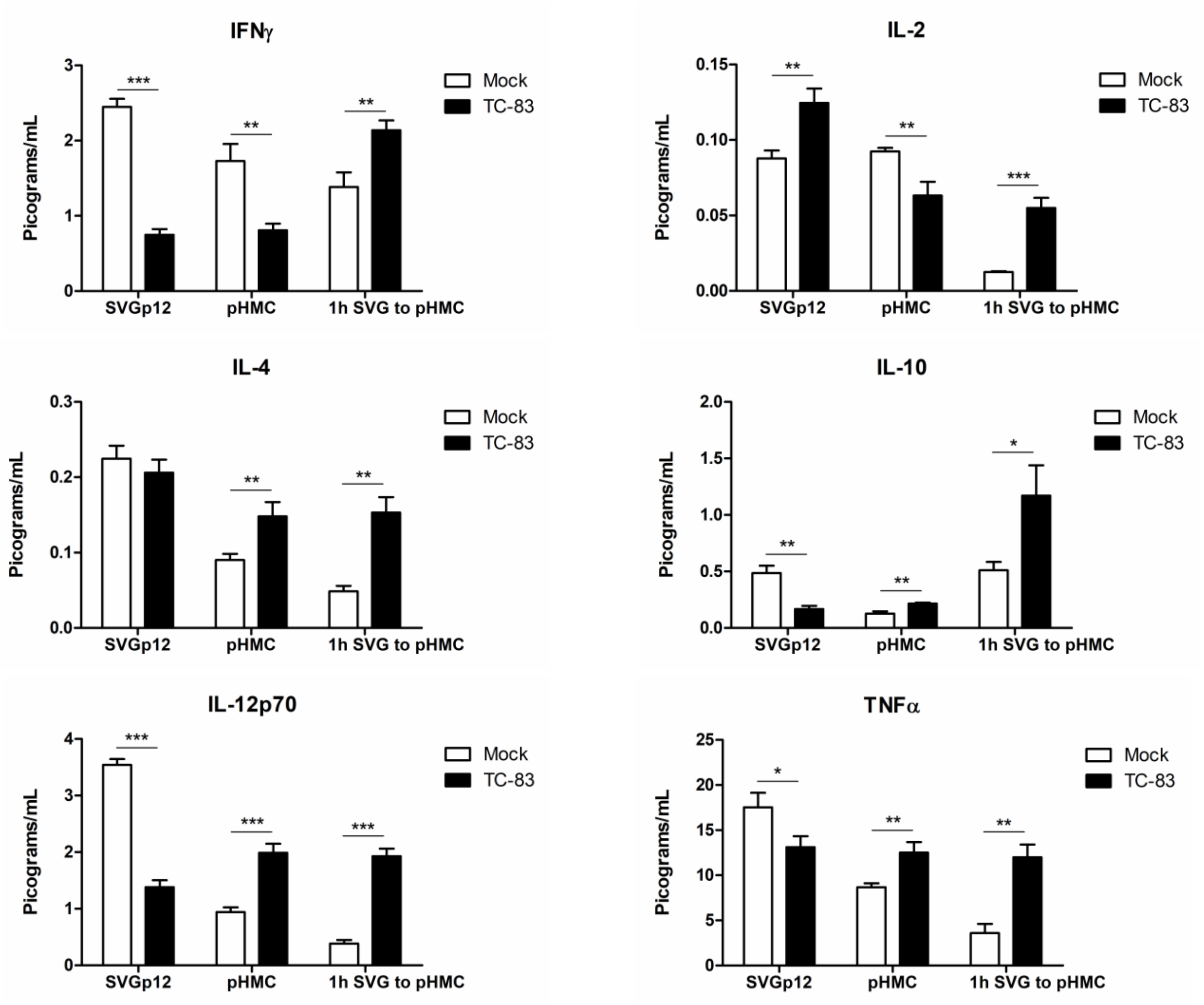


***Supplemental Figure 3.*** TC-83 induced inflammation in primary human astrocyte (SVGp12) and primary human microglial cells (pHMC). Inflammatory cytokine profiles are displayed for directly infected TC-83 (MOI:2) infected SVGp12 and pHMC cells at 2hpi, and pHMC cells receiving 1hpi SVGp12 supernatants for 2h. The quantitative data are depicted as the means of six biologically independent experiments ± SD. * $p < 0.05$; ** $p < 0.01$; *** $p < 0.001$.
